# Genomic Analysis of Multidrug-Resistant *Escherichia coli* Strains Isolated in Tamaulipas, Mexico

**DOI:** 10.3390/tropicalmed8100458

**Published:** 2023-09-26

**Authors:** Jessica L. Ortega-Balleza, Abraham Guerrero, Graciela Castro-Escarpulli, Ana Verónica Martínez-Vázquez, María Antonia Cruz-Hernández, Erick de Jesús de Luna-Santillana, Erika Acosta-Cruz, Irám Pablo Rodríguez-Sánchez, Gildardo Rivera, Virgilio Bocanegra-García

**Affiliations:** 1 Instituto Politécnico Nacional, Centro de Biotecnología Genómica, Boulevard del Maestro SN esq. Elías Piña, Col. Narciso Mendoza, Reynosa 88710, Mexico; jessica_ortega7@hotmail.com (J.L.O.-B.); avmartinez07@gmail.com (A.V.M.-V.); tonitacruz@gmail.com (M.A.C.-H.); edeluna@ipn.mx (E.d.J.d.L.-S.); gildardors@hotmail.com (G.R.); 2 CONACyT Program, Centro de Investigación en Alimentación y Desarrollo, Mazatlán 82112, Mexico; aguerrero@ciad.mx; 3 Instituto Politécnico Nacional, Escuela Nacional de Ciencias Biológicas, Ciudad de Mexico 07738, Mexico; chelacastro@hotmail.com; 4 Departamento de Biotecnología, Facultad de Ciencias Químicas, Universidad Autónoma de Coahuila, Saltillo Coahuila 25280, Mexico; erika.acosta@uadec.edu.mx; 5 Laboratorio de Fisiología Molecular y Estructural, Facultad de Ciencias Biológicas, Universidad Autónoma de Nuevo León, Ave. Pedro de Alba s/n cruz con Ave. Manuel L. Barragán, San Nicolás de los Garza 66455, Mexico; iramrodriguez@gmail.com

**Keywords:** antimicrobial resistance, *Escherichia coli*, mobile genetic elements, whole genome sequencing, dissemination, ARGs, surveillance, *bla*_CTX-M-15_, IncF

## Abstract

The global spread of antimicrobial resistance genes (ARGs) is a major public health concern. Mobile genetic elements (MGEs) are the main drivers of this spread by horizontal gene transfer (HGT). *Escherichia coli* is widespread in various environments and serves as an indicator for monitoring antimicrobial resistance (AMR). Therefore, the objective of this work was to evaluate the whole genome of multidrug-resistant *E. coli* strains isolated from human clinical, animal, and environmental sources. Four *E. coli* strains previously isolated from human urine (n = 2), retail meat (n = 1), and water from the Rio Grande River (n = 1) collected in northern Tamaulipas, Mexico, were analyzed. *E. coli* strains were evaluated for antimicrobial susceptibility, followed by whole genome sequencing and bioinformatic analysis. Several ARGs were detected, including *bla*_CTX-M-15_, *bla*_OXA-1_, *bla*_TEM-1B_, *bla*_CMY-2_, *qnrB*, *catB3*, *sul2*, and *sul3*. Additionally, plasmid replicons (IncFIA, IncFIB, IncFII, IncY, IncR, and Col) and intact prophages were also found. Insertion sequences (ISs) were structurally linked with resistance and virulence genes. Finally, these findings indicate that *E. coli* strains have a large repertoire of resistance determinants, highlighting a high pathogenic potential and the need to monitor them.

## 1. Introduction

Antimicrobial resistance (AMR) represents one of the most urgent public health problems [1,2]. Excessive and inappropriate use of antimicrobials has led to the emergence of resistant bacteria and their subsequent dissemination among bacteria in different environments. AMR leads to antimicrobial treatment failure in both humans and animals [3]. In 2019, infections due to multidrug-resistant (MDR) bacteria were responsible for 1.27 million deaths mainly attributed to *Escherichia coli* (*E. coli*) [1].

Due to its ubiquity and genomic plasticity, *E. coli* [4] is one of the main bacteria involved in the spread of AMR in communities, foods, farms, animals, the environment, and in clinical settings [5] since it can constantly exchange external genetic material such as ARGs through mobile genetic elements (MGEs) [3,6]. Thus, this bacterium is used as an excellent index not only to monitor AMR but also prevalence, types, and movement of ARGs [5,7].

*E. coli* represents a threat to public health due to AMR and the versatility of pathotypes, which lead to intestinal and extraintestinal infections [7,8]. Extraintestinal pathogenic *E. coli* (ExPEC) strains are opportunists that remain asymptomatically in the intestine to subsequently colonize extraintestinal sites [9].

Previous studies [10,11,12] in the Tamaulipas state have evidenced by molecular techniques the circulation and high prevalence of MDR *E. coli* strains among different environments. However, the MGEs responsible for the spread of AGRs have not been investigated and consequently, it is unknown what elements are involved in the dispersal of MDR in the region, as well as the genetic diversity they present.

The implementation of WGS is an alternative to improve surveillance of MDR pathogens of health concern by overcoming the limitation of analyzing only a small part of the genome and providing faster management and monitoring of the emergence of new antibiotic-resistant strains and their evolution [13].

Therefore, in support of the One-Health program as well as the health concerns generated by antimicrobial resistance, the objective of this work was to evaluate whole genome multidrug-resistant *E. coli* strains from human clinical and environmental sources as well as to characterize the mobilome and resistome that contribute to the spread of antimicrobial resistance genes.

## 2. Materials and Methods

### 2.1. E. coli Strains

*E. coli* strains used in this study are part of a bacterial collection from the Environment-Microorganism Interaction Laboratory of the Centro de Biotecnología Genómica of the Instituto Politécnico Nacional and are also part of a larger project on antimicrobial resistance (Table 1). Two *E. coli* strains were collected from routine nosocomial pathogen testing specimens at a tertiary care hospital in Reynosa, Tamaulipas, Mexico. No patient data were requested. Therefore, no institutional review board (IRB) approval or informed consent was required.

### 2.2. Isolation of Strains

*E. coli* strains (n = 4) were collected between 2015 and 2018 and were previously identified by standard biochemical tests (lactose, indole, methyl red, Voges–Proskauer, Simmons Citrate, Christensen’s urea, and H_2_S production). The strains were grown in TSA (MCD LAB) and EMB (Eosin Methylene Blue) medium (MCD LAB) at 37 °C for 24 h. In addition, they were confirmed by ChromAgar orientation.

### 2.3. Antimicrobial Susceptibility Testing

Antimicrobial susceptibility analysis was performed using the disk diffusion method, following established guidelines [14]. Sixteen antibiotics were tested: tetracycline (TET, 30 µg), doxycycline (DOX, 30 µg), minocycline (MIN, 30 µg), ampicillin (AMP, 10 µg), amoxicillin/clavulanic acid (AMC, 20/10 µg), ciprofloxacin (CIP, 5 µg), trimethoprim-sulfamethoxazole (SXT, 25 µg), levofloxacin (LEV, 5 µg), streptomycin (STR, 30 µg), gentamicin (GE, 10 µg), cephalothin (CF, 30 µg), cefepime (FEP, 30 µg), cefotaxime (CTX, 30 µg), amikacin (AK, 30 µg), ceftriaxone (CRO, 30 µg), and chloramphenicol (CHL, 30 µg). *E. coli* ATCC 25,922 was used as a quality control strain.

### 2.4. Whole Genome Sequencing

For genomic DNA extraction, the strains were grown in LB broth (Condalab, Madrid, Spain) under agitation at 37 °C for 24 h. Subsequently, DNA was obtained using the Promega Wizard Genomics extraction kit (Promega Corp., Madison, WI, USA) and QIAmp^®^ DNA Mini Kit (QIAGEN, Hilden, Germany). Whole genome sequencing was performed at the National Laboratory of Animal Digestive Nutrigenomics and Microbiomics (LANMDA-IPN) and DNA quantification was performed using the Qubit dsDNA HS Assay kit on the Qubit 3.0 fluorometer (Thermo Scientific, Waltham, MA, USA). Libraries were constructed using the Nextera Flex library kit. Libraries were sequenced using the MiniSeq™ sequencing system (150 bp paired-end reads).

### 2.5. Bioinformatic Analysis

Reads quality was assessed through FastQC v0.11.3 (https://www.bioinformatics.babraham.ac.uk/projects/fastqc/, accessed on 10 October 2021). Cleaning of raw reads was performed with Trim Galore v0.6.6 (https://www.bioinformatics.babraham.ac.uk/projects/trim_galore/, accessed on 12 October 2021). Assembly was performed using SPAdes v3.15.2 with settings —isolate and –k 21,31,41,51,61,71,81,91. The quality of the assemblies was assessed with QUAST v5.0.2 (https://github.com/ablab/quast, accessed on 12 October 2021). Contigs smaller than 500 bp were removed. Sequences were deposited in GenBank under bioproject PRJNA749581. Moreover, 31HGR *E. coli* strain was previously published as a draft genome [15] and it has been deposited in GenBank under the accession number JAKJKJ000000000.

Automatic annotation was performed in Rapid Annotation using Subsystem Technology from the Pathosystems Resource Integration Center (PATRIC) v3.6.9 [16] (now Bacterial and Viral Bioinformatics Resource Center https://www.bv-brc.org/, accessed on 15 October 2021). Manual annotation was executed in the Center for Genomic Epidemiology (https://www.genomicepidemiology.org/, accessed on 1 December 2021) bioinformatics tools such as ResFinder v4.1 (https://cge.food.dtu.dk/services/ResFinder/, accessed 1 December 2021), PlasmidFinder v2.1 (https://cge.food.dtu.dk/services/PlasmidFinder/, 1 December 2021), and SerotypeFinder v.2.0 (https://cge.food.dtu.dk/services/SerotypeFinder/, 3 December 2021). Other MGEs such as ISs and phage were analyzed by ISSaga v2.0 (https://issaga.biotoul.fr/, accessed on 2 December 2021) and PHASTER (https://phaster.ca/, accessed on 3 December 2021) [17], respectively. ISs that could not be identified by ISSaga were searched by BLASTn. Sequence types (STs) were determined in silico using the PubMLST database (https://pubmlst.org/, accessed on 3 January 2022). Default parameters were used for all software unless otherwise specified.

### 2.6. Phylogenomic Analysis and Genomic Comparison

Using the Similar Genome Finder of PATRIC web resources v3.6.12, a search for close reference genomes was performed. The sequences of these genomes, in FASTA format, were deposited in the CSIPhylogeny v1.4 pipeline (https://cge.cbs.dtu.dk/services/CSIPhylogeny/, accessed on 6 April 2022). Default parameters were used: minimum depth at SNP positions: 10×; minimum relative depth at SNP positions: 10%, minimum distance between SNPs: 10 bp; minimum SNP quality: 30; minimum read mapping quality: 25, and minimum Z-score: 1.96. *E. coli* K12 substr. MG1655 (GenBank: U00096.3) was used as the reference strain. Subsequently, to infer phylogeny from SNPs, the program MEGA X v10.0.5 [18] was employed using the maximum likelihood method and based on the Tamura–Nei model. Genomic comparisons were performed in GView Server v3.0 (https://server.gview.ca/, accessed on 29 April 2023) in which the four genomes that were sequenced in this study were combined with a few related genomes based on the data obtained from the Similar Genome Finder. The reference genome used was *E. coli* K12 substr. MG1655 (Genbank: U00096.3). The genomic context of regions containing resistance genes was analyzed using EasyFig v2.2.2 [19].

OrthoVenn2 web server [20] (https://orthovenn2.bioinfotoolkits.net/home, accessed on 28 April 2023) was used to predict orthologous gene clusters among the *E. coli* strains and default parameters were used.

## 3. Results

### 3.1. Antibiotic Susceptibility

Phenotypic resistance was identified mainly to TET, AMP, STR, SXT, CHL, and GE (Table 2). All four strains exhibited resistance to TET, while resistance to AMP was present in three strains as well as STR, SXT, CHL, and GE. Quinolone resistance was observed in *E. coli* 31HGR, 47C, and 87CLU. All *E. coli* strains were identified as MDR.

### 3.2. Genomic Characteristics

The total genome size on average ranged between 4.72 Mb and 5.31 Mb, and the GC content was found to be between 50.80% and 50.95%. The number of contigs and N50 values are described in Table 3.

### 3.3. Resistome

All strains had multiple acquired antibiotic resistance genes. Aminoglycoside resistance genes included *aadA1*, *aadA2*, *aadA5*, *aac3-IIa*, *aph6-Id*, *aph*(*3*″)*-Ia*, *aph*(*3*″)-*Ib*, and *aac*(*6*’)-*Ib-cr*. Resistance genes to β-lactams (*bla*_OXA-1_, *bla*_TEM-1B_, *bla*_CMY-2_, and *bla*_CTX-M-15_), sulfonamides (*sul1*, *sul2*, and *sul3*), trimethoprim (*dfra12*, *drfa14,* and *drfa17*), phenicols (*catB3*, *cmlA1,* and *floR*), tetracyclines (*tet*(*A*), and *tet*(*B*)), quaternary ammonium compounds (QCA) (*qacE* and *qacL*), macrolides (*mdfA* and *mphA*), quinolones (*qnrB*), and bacitracin (*bacA*) were also found (Table 4, Tables S1 and S2).

The *tet*(*A*), *aadA2*, *aph*(*3*″)-*Ib*, and *aph*(*6*)*-Id* genes were the most common among the genomes. The clinic strain 31HGR was found to have the *bla*_CTX-M-15_ and *bla*_OXA-1_ genes, among others. Additionally, gene *qnrB* was found in the surface water isolate (3AS) of the Rio Grande/Rio Bravo River. The *sul2* and *sul3*, *bla*_TEM-1B_, and *bla*_CMY-2_ genes were the most relevant resistance genes identified in 87CLU and 47C.

Quinolone resistance was detected by mutations in three genes: *gyrA*, *parC*, and *parE*. Two mutations were found in *gyrA* (S83L, D87N), one mutation in *par*C (S80I), and two mutations in *parE* (S458T, S458A). All mutations in *gyrA* and *parC* were present in strains 31HGR, 47C, and 87CLU. The *parE* (S458T) mutation was identified only in strain 31HGR, while the *parE* (S458A) mutation was present in strains 87CLU and 47C. Additionally, strain 3AS *E. coli* did not exhibit any of these mutations (Table 5).

### 3.4. Mobilome of E. coli

#### 3.4.1. Plasmids

Multiple plasmid replicons were identified in all genomes (Table 4). The conjugative IncFIB replicon was the most prevalent in all strains. IncFIA and IncFII replicons were detected in 31HGR, 87CLU, and 47C. Meanwhile, IncY (phage-like plasmid) was present in 87CLU and 47C. The mobilizable replicons IncR and Col were identified in 3AS. All genomes evaluated had four plasmids.

#### 3.4.2. Phages

A total of 18 intact prophages were identified; strain 47C harbored the most elements of this type (seven), followed by 87CLU which had five, while 31HGR had four and 3AS harbored three (Table 6). All the prophages encoded mainly hypothetical and structural proteins, except for the *bla*_CMY-2_ gene found exclusively in the IncY phage-like plasmid of 47C and 87CLU. In addition, IncY was found as an intact prophage in 47C *E. coli* strains.

#### 3.4.3. Other EGMs

All strains possessed the *int*I1 gene, which codes for the integrase of the class 1 integron. Due to the characteristics of the reads, only the *int*I1 gene was recovered intact and adjacent to its resistance gene cassette in strains 31HGR and 3AS, while in strains 47C and 87CLU, it was found in separate contigs.

Moreover, multiple ISs associated with resistance or virulence genes were detected (Table S3); IS91 was detected close to *aph*(*6*)-*Id*, *tet*(*A*), *s*u*l2*, and *fl*o*R*. ISEcp1 was detected adjacent to *bla*_CTX-M-15_ and *bla*_CMY-2_. IS26 was also identified by BLASTn adjacent to resistance genes (Table S4).

Common co-resistance patterns were detected in 47C and 87CLU, which have the arrangement *sul2::IS91::floR::IS91* and *IS91::floR::IS91::IS91::sul2*, respectively, where the difference is in the position of genes involved in resistance to sulfonamides and phenicols. Furthermore, in these two strains, the *aadA1::aadA2:cmlA:aadA1:qacL:IS256* arrangement in 87CLU is very similar to the *aadA2:cmlA:aadA1:qacL:IS256:sul3* of 47C. These arrangements confer co-resistance to aminoglycosides, chloramphenicol, quaternary ammonium compounds, and sulfonamides.

### 3.5. Virulome

A total of 18 virulence-associated genes were identified (Table 4). The strains belonging to ST224 had the highest number of these genes (14), 31HGR had 12, and 3AS had the lowest number of genes (6). The *csg*, *fimH*, *ompA*, and *hlyE* genes were identified in all four strains, the *iutA*, *iucA*, and *sitA* genes were common in *E. coli* 31HGR, 47C, and 87CLU, which harbored the highest number of resistance genes. Genes from type II, III, IV, and VI secretion systems were also identified.

### 3.6. Phylogenomic Analysis

**Figure 1:**
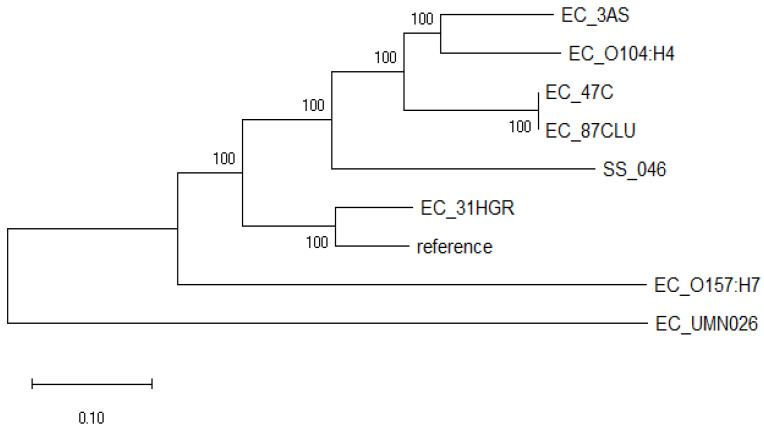
Phylogenomic tree of *E. coli* strains sequenced in this work (31HGR, 87CLU, 47C, and 3AS) and close reference genomes based on single nucleotide polymorphism (SNPs) differences and constructed with the maximum likelihood method. Evolutionary distances were calculated using the Tamura–Nei model at 100 replicates.

This analysis showed that the *E. coli* strains evaluated diverged into two well-defined clades (Figure 1). One clade consisted only of EC_UMN026 (CU928163.2), an MDR uropathogenic *E. coli*. On the other hand, the second clade was subdivided into two clearly differentiated groups: one comprised only by shigatoxigenic *E. coli* EC_O157:H7 (BA000007.2), and the other consisted of the strains sequenced in this study and those previously reported EC_O104:H4 (CP003289.1), EC_ K12 (U00096.3) and SS_046 (CP000038.1); 31HGR showed a divergence from the rest of the strains as it grouped only with the reference strain. Meanwhile, 3AS exhibited a close relationship with the intestinal pathogen EC_O104:H4 (CP003289.1).

### 3.7. Comparative Genomics

**Figure 2:**
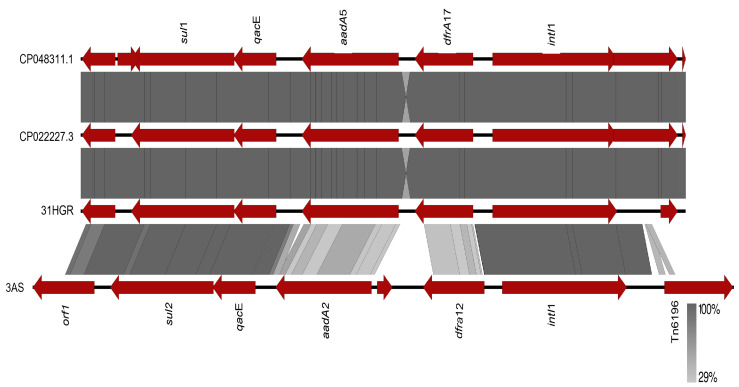
Comparison of the genomic environment of the intI1 gene of reported plasmids CP048311.1 and CP022227.3 against the sequences of *E. coli* strains 31HGR and 3AS. The gray shaded region between the sequences indicates the similarity according to BLASTx (29–100%).

The genomic environment of the class 1 integron resistance gene cassettes of strains 31HGR and 3AS, which had the most resistance gene cassettes in the same contig, was analyzed (Figure 2). The comparison was performed against the sequences of p32-4_A (CP048311.1) and pCTXM15_000200 (CP022227.3).

A 100% similarity was observed between *E. coli* 31HGR and p32-4_A (CP048311.1) and pCTXM15_000200 (CP022227.3), whereas 3AS presented a lower similarity since, although they have the same resistance gene families, they have different variants.

**Figure 3:**
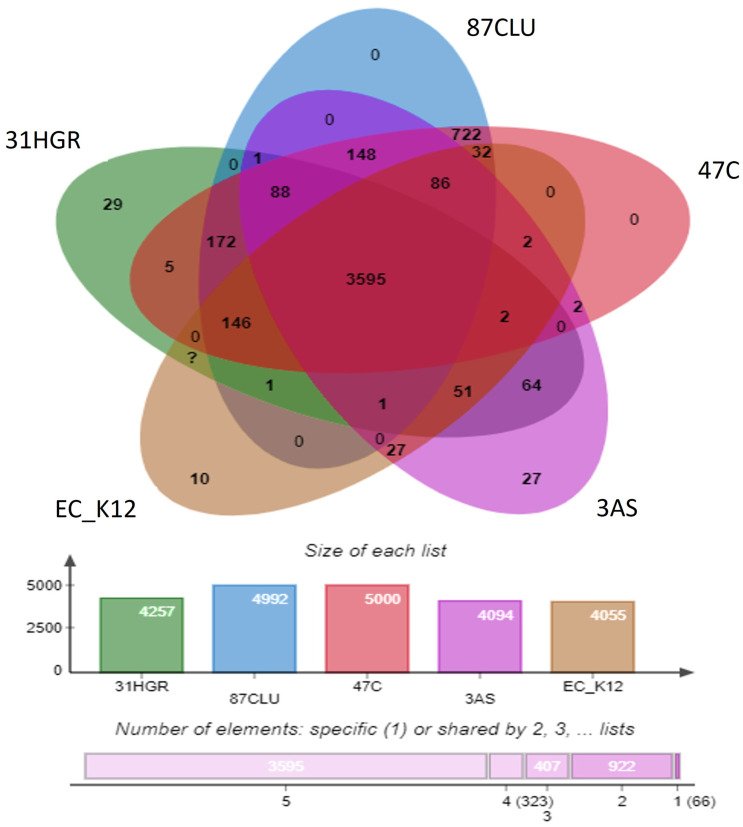
Venn diagram of orthologous gene clusters present in *E. coli* strains 31HGR, 87CLU, 47C, 3AS, and the reference strain.

Orthologous gene analysis identified that strain 31HGR contained 4686 proteins and 4257 clusters, 5046 proteins and 5000 clusters in 47C, 5047 proteins and 4992 in 87CLU, and 4477 proteins and 4094 clusters; the reference strain EC_ K12 (U00096.3) consisted of 4305 proteins and 4055 clusters. In addition, among the orthologous gene clusters, the core genome consisted of 2595 clusters (Figure 3). The species formed 5313 clusters, 1741 orthologous clusters, and 3572 single-copy gene clusters. Strains 87CLU and 47C shared the most gene clusters and these bacteria belong to the same ST. Strains 31HGR and 3AS had the most single-copy gene clusters (357).

**Figure 4:**
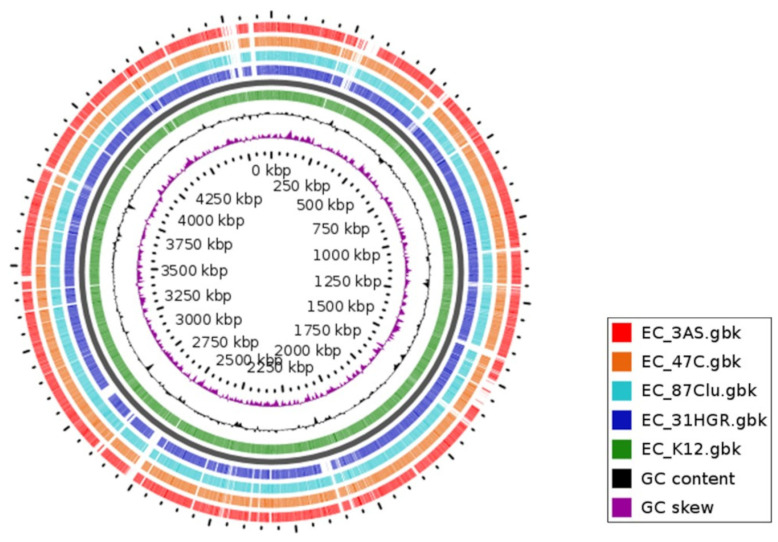
Comparative analysis of *E. coli* genomes with the reference strain. The BLAST atlas was obtained using GView Server. Blanks indicate areas where there is no similarity. Colored rings match the color of the strains. *E. coli* K12 (U00096.3) was used as a reference genome.

The GView analysis showed the similarity between the different genomes sequenced in the present work. It is also possible to visualize the variations in GC content in different regions; those areas that are not shared with those of the reference genome show a higher content with respect to the genome average, which is evidence of genetic material acquired by HGT (Figure 4).

## 4. Discussion

The findings of this work show a diversity of the resistome, mobilome, and virulome of *E. coli* strains isolated from different sources in Tamaulipas. Most of the ARGs were detected associated with the class 1 integron or ISs. To our knowledge, this is the first study that provides a genomic evaluation of antimicrobial resistance and MGEs in *E. coli* strains in northeastern Tamaulipas.

In this work, resistance to antibiotics that are currently of clinical relevance such as quinolone resistance mediated by chromosomal mutations in the *gyrA* (S83L, D87N), *parC* (S80I), and *parE* (S458T, S458A) genes were detected in the genomes of *E. coli* from human isolates and retail meat. These findings correlate with the phenotypic resistance observed to ciprofloxacin, levofloxacin, and nalidixic acid, which are relevant antibiotics for the treatment of a wide range of infections, mainly in urinary tract infections (UTIs) [21]. In addition, plasmid-mediated quinolone resistance (PMQR) was also identified by the presence of *qnr*B and *aac*(*6*’)-*Ib-cr* genes. These genes by themselves confer low levels of resistance [22]; in this work, the 3AS strain harboring *qnr*B did not exhibit resistance to quinolones, which is consistent with that previously reported [23], and also did not present chromosomal mutations that confer resistance to this family.

Aminoglycoside resistance genes were common; *aph*(*6*)-*Id* and *aph*(*3*″)-*Ib* were present in three strains (31HGR, 87CLU and 47C) both encoding phosphotransferases. However, only 87CLU and 47C exhibit phenotypic resistance to two members of this antibiotic family: gentamicin and streptomycin, which still have clinical relevance against Gram-positive and Gram-negative infections, as they act synergistically with β-lactams and glycopeptides [24].

Regarding resistance to β-lactams, *bla*_OXA-1_, *bla*_CTX-M-15_, *bla*_TEM-1B_, and *bla*_CMY-2_ genes were presented in this study of which CTX-M-15 is the most prevalent and globally distributed ESBL in *E. coli* [25] and is associated with UPEC ST131 [26].

Furthermore, *bla*_CMY-2_ encodes for a plasmid-mediated AmpC-type β-lactamase [23] that confers resistance to all β-lactams except the fourth generation of extended-spectrum cephalosporins and carbapenems [27]. These β-lactamases are more concerning than ESBLs because they are typically resistant to inhibitors such as clavulanic acid, tazobactam, and sulbactam, thereby becoming clinically relevant [5,28]. Moreover, the combination of ESBL and AmpC genes with the loss of outer membrane porin can cause resistance to carbapenems [27].

Due to its spread by plasmids from different groups, the *bla*_CMY-2_ gene is commonly found in *E. coli* worldwide from human, animal, and environmental sources [29]. In North America, this gene is commonly found in *E. coli* from cattle [30] Additionally, it has been previously identified in pediatric patients in Mexico [31]. However, in this study, *bla_CMY-2_* was identified not only in retail meat (47C) but also in *E. coli* that was isolated from humans (87CLU), emphasizing the transmission of *bla_CMY-_*_2_ from animals to humans.

Interestingly, PHASTER revealed that in the two strains harboring *bla*_CMY-2_, it was embedded in a phage-like plasmid, IncY; in both cases, *bla*_CMY-2_ was adjacent to ISEcp1, which explains its mobility by THG between plasmids and between bacteria from different environments.

In this work, the presence of the *mphA* gene in 31HGR also is noteworthy; although phenotypic resistance was not observed, its presence is of concern since macrolides are usually used mainly as therapeutics against Gram-positives [32]. Of this family of antibiotics, azithromycin has shown greater activity and offers an alternative treatment against *Shigella, Salmonella*, and enterotoxigenic *E. coli* (ETEC) [33,34]. Therefore, plasmid-mediated macrolide resistance is alarming; *mphA* is the most relevant resistance mechanism since it has been observed to increase MIC in *E. coli*, and another aspect of concern is that *E. coli* is serving as a reservoir for this gene and could transfer it to clinically important bacteria [5,33].

Additionally, all *E. coli* strains that exhibited phenotypic resistance to tetracyclines harbored the *tet*(*A*) gene, *tet*(*B*) gene, or both. Notably, the *tet*(*A*) gene is readily dispersed between different environments [35] which is largely because such a gene is frequently harbored by plasmids of a wide host range and a different incompatibility group [36].

On the other hand, in this study, the genes for resistance to sulfonamides, trimethoprim-sulfamethoxazole and aminoglycosides, were identified forming part of the class 1 integron, which is congruent since these cassettes are frequently associated with such MGEs in commensal and pathogenic *E. coli* from animal, environmental, and human sources [37,38].

Currently, an increase in phenotypic resistance to multiple antibiotics has been reported in different types of sequences around the world and obtained from different environments; this situation can lead to therapeutic failures. In this work, it was detected that *E. coli* 31HGR belongs to ST44, which is part of CC10, as is ST131, and is well known for being opportunistic, MDR, and associated with intestinal and extraintestinal infections in both humans and animals [9]. Whereas 47C and 87CLU belong to ST224, which is considered pandemic and related to MDR, mainly to β-lactams and carbapenems [39], in this work, the two ST224 harbored *bla*_TEM-1B_ and *bla*_CMY-2_ genes. Similarly, this study identified ST155 in *E. coli* strain 3AS, which originates from animals and is linked to the transmission of multidrug resistance (MDR) via plasmids [23,40].

It is noteworthy that the *E. coli* strains 47C and 87CLU, which belong to the same ST and have a similar genomic content, showed different phenotypic resistance profiles; this situation may be influenced by the fact that the bacteria were isolated from different sources (human and retail meat), so the exposure to selective pressures in their environment are different and could influence the expression of resistance genes.

Regarding phenotypic quinolone resistance, here, 87CLU and 47C had a different profile. Zhang et al. showed that isolates with the combination S83L + D87N mutation had resistance to statistically different drugs between ciprofloxacin and levofloxacin [41]. Resistance to quinolones is a major concern because they are some of the most potent classes of antibiotics that are currently available. Consequently, the World Health Organization (WHO) has prioritized combating quinolone resistance [42].

In addition, different plasmid replicons were also identified in the present study with IncF being the most common; this element is widely dispersed in Enterobacterales and is known to be epidemic and to harbor ARGs such as *bla*_CTX-M-15_ in addition to virulence genes [43]. Interestingly, in strain 3AS, the Col-like mobilizable replicon, a small 6 to 10 Kb, colicin-producing plasmid also associated with the spread of *qnr* family genes, was detected, which is consistent with the findings reported here, as *qnr*B was identified in strain 3AS [44].

Multiple transposable elements were identified; in some cases, they were found adjacent or close to ARGs, which raises concern for their ability to mobilize genetic material such as ISEcp1 of the IS1380 family related to the mobilization of a wide variety of ARGs, mainly β-lactam resistance [45] as identified in this work, adjacent to *bla*_CMY-2_ and *bla*_CTX-M-15_. In the present study, IS91 was among the most associated with antibiotic resistance genes, [5] and [46] reported similar results in *E. coli*; these types of sequences can mobilize adjacent genes through a single-end transposition process [46]. Detection of multiple insertion sequences and compound transposons flanking antibiotic resistance genes or located near them emphasizes the significance of these elements in mobilizing the genes within *E. coli* strains isolated in our region.

The spread and increase of clinically relevant UTIs in different surroundings is an alarming health and environmental concern [47]. In Mexico, treatment of urinary tract infections generally involves trimethoprim with sulfamethoxazole, quinolones, second- and third-generation cephalosporins, nitrofurantoin, and fosfomycin [48,49]. In our work, resistance to some of the first-choice treatment antibiotics was found, which is of concern because such strains could successfully establish in different environments and subsequently lead to difficult-to-treat infections. In Mexico, similar findings were reported in UPEC [22,38,50].

Moreover, numerous virulence genes were identified, especially those related to iron uptake. Notably, the strain 87CLU from phylogroup B1 harbored the highest number of virulence genes, so these findings are important because these bacteria and their clones could reach other environments closely related to humans.

Phylogenetic analysis showed a close relationship between *E. coli* strains regardless of the isolation site. The 31HGR, 47C, 87CLU, and 3AS genomes were related to previously reported strains that are of intestinal origins (either pathogenic or commensal) such as *E. coli* O157:H7 and *E. coli* 0104:H4. It is noteworthy that 31HGR, 47C, and 87CLU harbored genes that are common to ExPECs, especially 31HGR, which was the strain that exhibited divergence with respect to the other genomes sequenced in this work; this evidences the evolution of intestinal bacteria given the need to adapt to different environments [51] by acquiring genetic material from ExPECs.

Genomic analysis of *E. coli* MDR strains from different environments revealed a diversity of ARGs and virulence genes, as well as multiple MGEs involved in their propagation that confer to the bacterium advantages not only to colonize, but also to persist in different environments [38]. An important limitation of this study is the small number of sequenced isolates. Consequently, it does not offer a comprehensive overview of the wide range of clonal groups circulating within our area, including the ARGs and MGEs. In addition, the reconstruction of certain important MGEs, such as plasmids and their genomic content, is difficult due to the short reads. Therefore, larger-scale epidemiological studies are required to monitor multidrug-resistant bacteria and their MGEs to determine the possible routes and fates of ARGs.

## 5. Conclusions

Finally, the findings of this work evidence the circulation in the environment and in the community of bacteria with a diversity of antimicrobial resistance determinants and virulence genes, as well as multiple MGEs involved in their propagation, which represent a health concern because they could lead to therapeutic failures. Therefore, epidemiological monitoring is of great importance to know and control the spread of these MDR bacteria.

## Figures and Tables

**Table 1:** *E. coli* strains included in this study.

Isolate ID	City	Source	Year
31HGR	Reynosa	Urine (human)	2018
87CLU	Reynosa	Urine (human)	2018
47C	Rio Bravo	Chicken retail meat	2017
3AS	Diaz Ordaz	Surface water (Rio Grande River)	2018

**Table 2:** Phenotypic resistance profiles identified in *E. coli* strains.

Antimicrobial Family	Strains			
	31HGR	87CLU	47C	3AS
Tetracyclines	TET MIN DOX	TET	TET MIN DOX	TET DOX
Penicillins	AMP	AMP	AMC AMP	AMC
Aminoglycosides	-	STR GE	STR GE	STR GE
Folate pathway antagonists	SXT	SXT	-	SXT
Quinolones	LEV CIP	LEV NA	CIP	-
Phenicols	-	CHL	CHL	CHL
Cephalosporins	CTX FEP	CTX CRO	CF	CF
Nitrofuran	-	-	NF	-
MDR	+	+	+	+

TET, tetracycline; DOX, doxycycline; MIN, minocycline; STR, streptomycin; LEV, levofloxacin; FEP, cefepime; CF, cephalothin; GE, gentamicin; CTX, cefotaxime; SXT, sulfamethoxazole–trimethoprim; AMP, ampicillin; CRO, ceftriaxone; CHL, chloramphenicol; AMC, amoxicillin/clavulanic acid; CIP, ciprofloxacin; NA, nalidixic. -, not detected; +, detected.

**Table 3:** General genome features of *E. coli* strains.

Features	31HGR	87CLU	47C	3AS
Source	Urine	Urine	Meat	Water
Contigs	141	162	148	96
N50	91,860	206,030	205,584	175,845
Size (Mb)	4.98	5.31	5.31	4.72
GC content (%)	50.82	50.80	50.82	50.95
CDS	4969	4690	5406	4690
tRNA	73	78	83	78
rRNA	6	10	9	10
CRISPR repetition	22	13	44	13
CRISPR spacer	20	12	42	12
CRISPR arrays	2	1	2	1
Serotype	O101:H4	O10:H23	O10:H23	O21:H21
Sequence type (ST)	44	224	224	155

**Table 4:** Resistome, mobilome, and virulome profiles of *E. coli* strains.

Isolate	SRA Number	Phenotypic Resistance Profile	ARG	MLST	Virulence Genes	Plasmid Replicon
31HGR	SRR15258840	SXT, CTX, LEV, FEP, AMP, CIP, TET, MIN, DOX	*tet*(*B*), *bla_OXA-1’_ bla_CTX-M-15’_ aadA5* *aph*(*6*)-*Id*, *aph*(*3*″)-*Ib*, *aac*(*6*’)-*Ib-cr*, *sul1*, *sul2*, *dfra17*, *catB3*, *qacE*, *mdfA*, *mph*(*A*)	44	*iucA*, *iutA*, *sitA*, *irp1*, *irp2*, *fyuA*, *ybt*, *csg*, *fimH*, *ompA*, *traT*, *hlyE*	IncFIA, IncFIB, IncFII, IncY
87CLU	SRR25437123	CHL, SXT, CTX, LEV, AMP, STR, GE, NA, CRO, TET	*tet*(*A*), *bla_TEM-1B_*, *bla_CMY-2_*, *aadA1*, *aadA2*, *cmlA1*, *aac3-IIa*, *aph*(*6*)-*Id*, *aph*(*3*″)-*Ia*, *aph*(*3*″)-*Ib*, *sul2*, *sul3*, *dfra14*, *floR*, *qacL*, *mdf*(*A*)	224	*iucA*, *iutA*, *sitA*, *hra*, *csg*, *fimH*, *IpfA*, *ompA*, *cvaC*, *gad*, *espP*, *cma*, *traT*, *hlyE*	IncFIA, IncFIB, IncFII, IncY
47C	SRR25602185	STR, CF, GE, AMP, CHL, NF, AMC, CIP, TET, MIN, DOX	*tet*(*A*), *tet*(*B*), *bla_TEM1B_*, *bla_CMY_*_2_, *aadA1*, *aadA2*, *cmlA1*, *aac3-IIa*, *aph*(*6*)-*Id*, *aph*(*3*″)-*Ia*, *aph*(*3*″)-*Ib*, *sul2*, *sul3*, *dfra14*, *floR*, *qacL*, *mdfA*	224	*iucA*, *iutA*, *sitA*, *hra*, *csg*, *fimH*, *IpfA*, *ompA*, *cvaC*, *gad*, *espP*, *cma*, *traT*, *hlyE*	IncFIA, IncFIB, IncFII, IncY
3AS	SRR25645458	CF, GE, SXT, CHL, AMC, STR, TET, DOX	*tet*(*A*), *aadA2*, *sul1*, *dfra12*, *floR*, *qnrB*, *qacE*, *mdfA*	155	*csg*, *fimH*, *IpfA*, *ompA*, *gad*, *hlyE*	IncFIB, IncY, IncR, Col

TET, tetracycline; DOX, doxycycline; MIN, minocycline; STR, streptomycin; LEV, levofloxacin; FEP, cefepime; CF, cephalothin; GE, gentamicin; CTX, cefotaxime; SXT, sulfamethoxazole–trimethoprim; AMP, ampicillin; CRO, ceftriaxone; CHL, chloramphenicol; AMC, amoxicillin/clavulanic acid; CIP, ciprofloxacin; NA, nalidixic.

**Table 5:** Mutational and plasmid-mediated quinolone resistance in *E. coli* strains.

Isolate ID	Mutations			Plasmid-Mediated
	*gyrA*	*parE*	*parC*	*qnrB*
31HGR	S83L, D87N	S458T	S80I	ND
87CLU	S83L, D87N	S458A	S80I	ND
47C	S83L, D87N	S458A	S80I	ND
3AS	ND	ND	ND	Detected

ND: Not detected.

**Table 6:** Distribution of intact prophages and their detected *E. coli* strains.

Strain	Intact Prophage	Region Length	GC %	Total Proteins	Most Common Phage
31HGR	1	43	49.85	56	PHAGE_Escher_phiV10_NC_007804
	2	35.1	51.84	33	PHAGE_Escher_pro483_NC_028943
	3	45.3	50.15	53	PHAGE_Entero_mEp460_NC_019716
	6	33.5	52.45	36	PHAGE_Entero_lambda_NC_001416
87CLU	2	41.7	50.93	62	PHAGE_Entero_SfV_NC_003444
	4	31.7	50.67	32	PHAGE_Klebsi_4LV2017_NC_047818
	11	13.6	55.02	20	PHAGE_Entero_P88_NC_026014
	12	12.1	55.44	19	PHAGE_Entero_P88_NC_026014
	13	9.2	53.04	16	PHAGE_Escher_pro147_NC_028896
47C	1	41.7	50.93	61	PHAGE_Entero_SfV_NC_003444
	4	34.7	50.61	37	PHAGE_Klebsi_4LV2017_NC_047818
	7	39.3	49.74	38	PHAGE_Escher_pro147_NC_028896
	8	95.5	47.64	106	PHAGE_Salmon_SJ46_NC_031129
	9	31.2	51.27	32	PHAGE_Entero_fiAA91_ss_NC_022750
	10	12.2	55.43	19	PHAGE_Entero_P88_NC_026014
	11	11.6	55.86	18	PHAGE_Entero_P88_NC_026014
3AS	1	57.3	51.10	86	PHAGE_Erwini_vB_EhrS_59_NC_048198
	2	43.1	51.85	52	PHAGE_Shigel_SfII_NC_021857
	3	23.8	51.60	34	PHAGE_Klebsi_4LV2017_NC_047818

## Data Availability

All the analyzed data are included in this published article.

## Supplementary Materials

The following supporting information can be downloaded at: https://www.mdpi.com/2414-6366/8/10/458/s1, Table S1: ARGs identified by ResFinder; Table S2: ARGs identified by BV-BRC (PATRIC) in *E. coli* strains; Table S3: Insertion sequences; Table S4: Synteny ARG and IS.
