# Supplementary material for: Genomic Analysis of Multidrug-Resistant Escherichia coli Strains Isolated in Tamaulipas, Mexico
Source: Trop Med Infect Dis. 2023 Sep 26;8(10):458. doi: 10.3390/tropicalmed8100458 (PMC10610597; doi:10.3390/tropicalmed8100458)
Supplement: Supplementary file 1 [file tropicalmed-08-00458-s001.zip › Supplementary Tables/Table S4 Synteny_ARG-IS.pdf]

Strain	Synteny of ARG and IS
31HGR	Tn3::*bla*$_{CTX-M-15}$: :**ISEcp1**<br>*sul*1:*qac*EdeltaΔ:*aad*A5:*dfra*17:*intI*1:Tn21Δ<br>*cat*B3:*bla*$_{OXA-1}$:*aac*(6')-Ib-cr<br>**IS91**:*aph*6-Id:*aph*(3'')-Ib:*sul*2<br>**IS91**:::*tet*(A)-*tet*R<br>**IS6**:::*mph*A
87CLU	*sul*2::**IS91**::*flo*R::**IS91**<br>*aadA*1:: *aadA*2:*cml*A:*aadA*1:*qacL*:IS256<br>**IS26**::*tet*R:*tet*A::**IS26**<br>*sug*E:*blc*:*bla*$_{CMY-2}$
47C	*aad*A2*:cmlA:aad*A1:*qac*L:**IS256**:*sul*3<br>**IS1**::*tet*(A)<br>***IS26****::aac(3)-IIa*<br>*sug*E:*blc*:*bla*$_{CMY-2}$:**ISEcp1**<br>**IS91**::*flo*R::IS91::**IS91**::*sul*2<br>*dfr*A14:**IS26**<br>*bla*$_{TEM-1}$*::**Tn2***
3AS	*sul2*:*qac*Edelta1:*aad*A2::*dfra*12:*intI*1:**Tn6196**<br>**IS91**::*flo*R::*tet*(A):tetR
