# Supplementary figures and images for: Genomic Analysis of Multidrug-Resistant Escherichia coli Strains Isolated in Tamaulipas, Mexico
Source: Trop Med Infect Dis. 2023 Sep 26;8(10):458. doi: 10.3390/tropicalmed8100458 (PMC10610597; doi:10.3390/tropicalmed8100458)

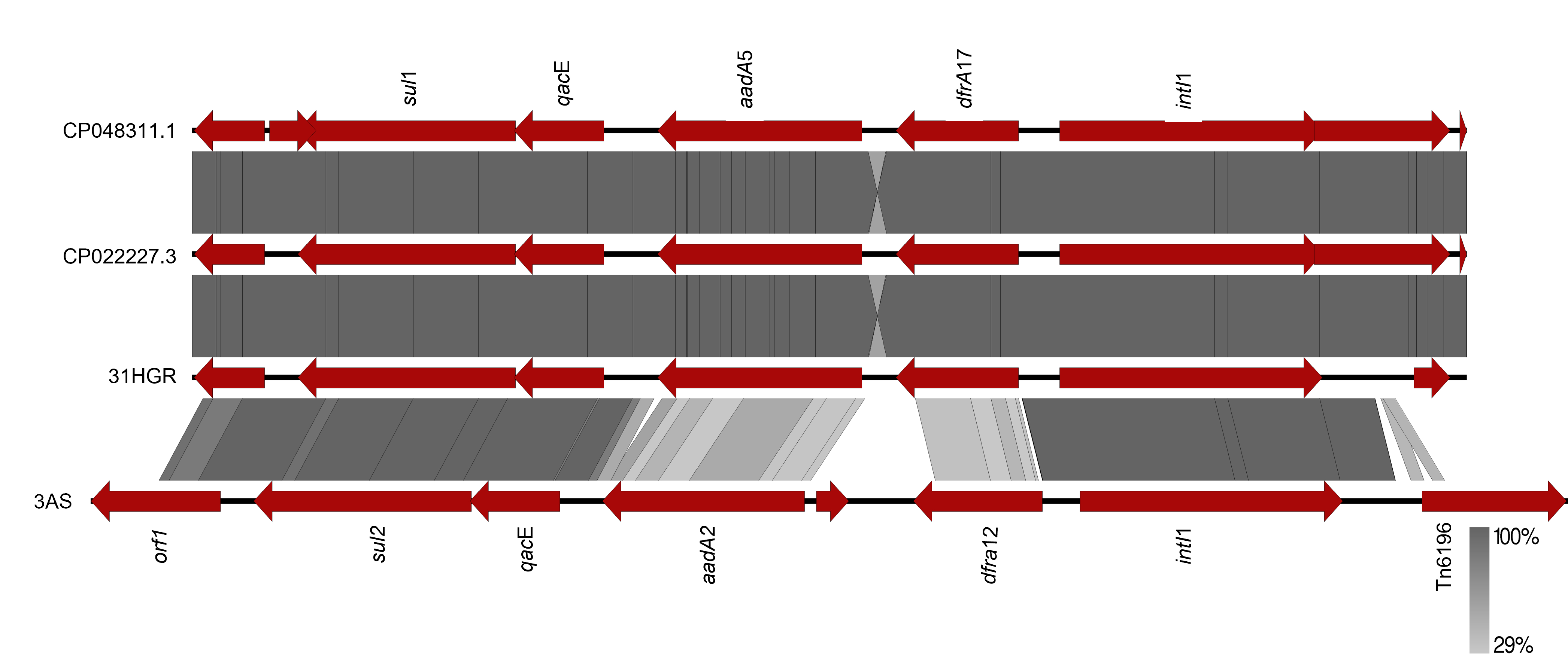

Supplement: Supplementary file 1 [file tropicalmed-08-00458-s001.zip › Figures/Comparative.jpg]

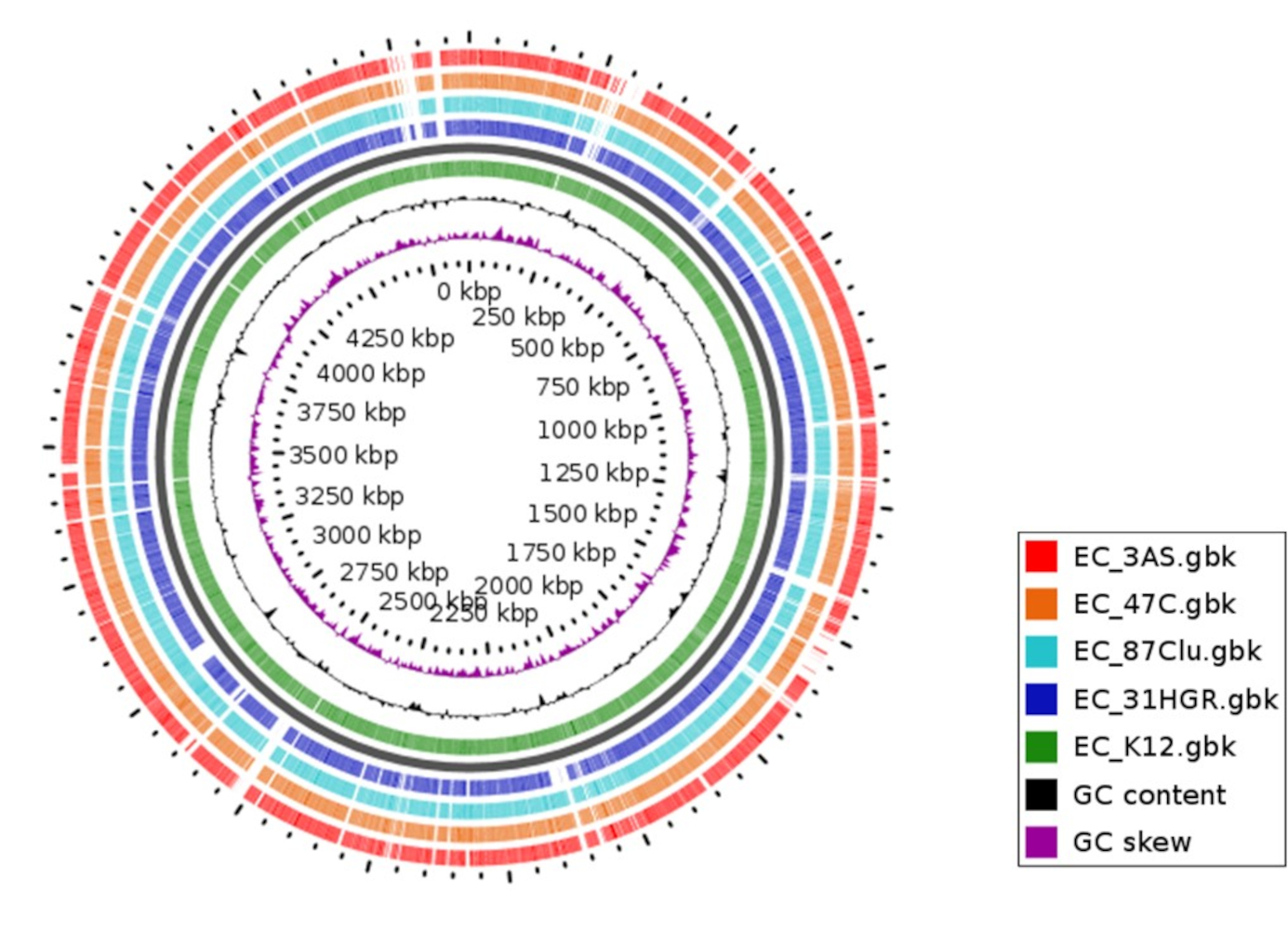

Supplement: Supplementary file 1 [file tropicalmed-08-00458-s001.zip › Figures/GView_Comparation_M.jpg]

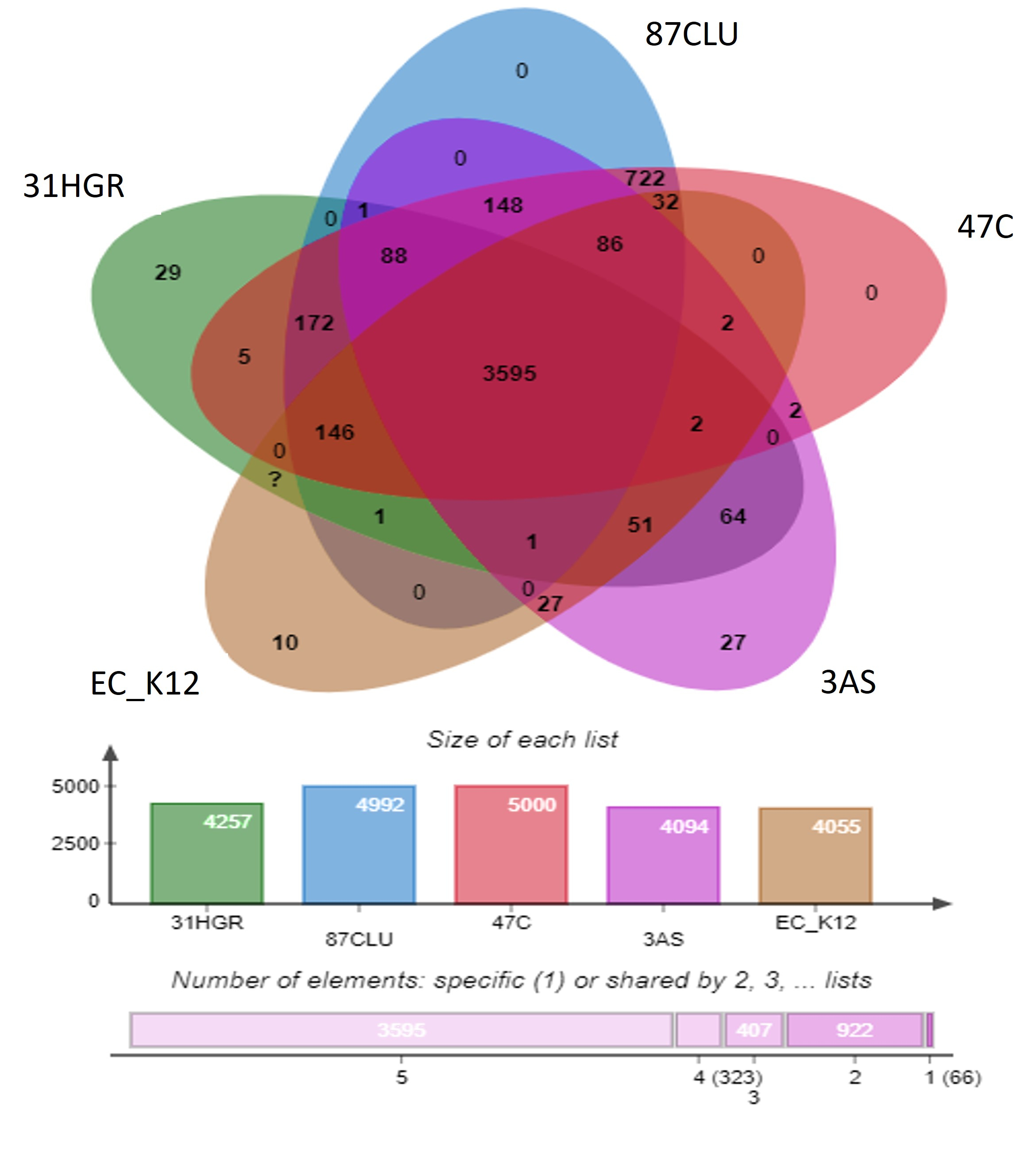

Supplement: Supplementary file 1 [file tropicalmed-08-00458-s001.zip › Figures/OrthoVen_results-.jpg]

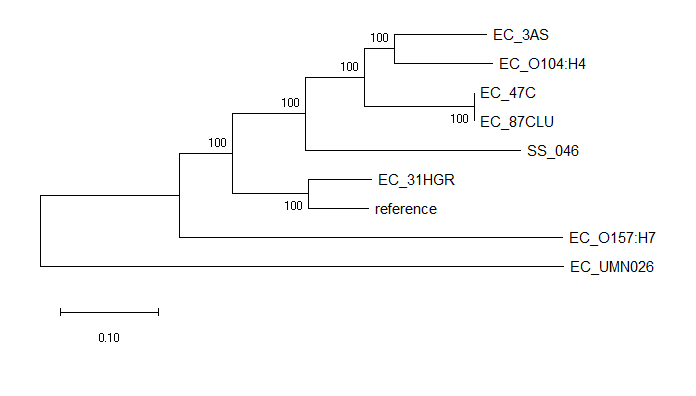

Supplement: Supplementary file 1 [file tropicalmed-08-00458-s001.zip › Figures/Phylogenetic_tree_paper.jpg]
